# Right vs. left ventricular longitudinal strain for mortality prediction after transcatheter aortic valve implantation

**DOI:** 10.3389/fcvm.2023.1252872

**Published:** 2023-09-07

**Authors:** Neria E. Winkler, Shehab Anwer, Kelly A. Reeve, Jonathan M. Michel, Albert M. Kasel, Felix C. Tanner

**Affiliations:** ^1^ Department of Cardiology, University Heart Center, University Hospital Zurich and University of Zurich, Zurich, Switzerland; ^2^ Department of Biostatistics, Epidemiology, Biostatistics and Prevention Institute, University of Zurich, Zurich, Switzerland

**Keywords:** aortic stenosis, transcatheter aortic valve implantation, global longitudinal strain, speckle tracking echocardiography, mortality

## Abstract

**Introduction:**

This study aims at exploring biventricular remodelling and its implications for outcome in a representative patient cohort with severe aortic stenosis (AS) undergoing transcatheter aortic valve implantation (TAVI).

**Methods and results:**

Pre-interventional echocardiographic examinations of 100 patients with severe AS undergoing TAVI were assessed by speckle tracking echocardiography of both ventricles. Association with mortality was determined for right ventricular global longitudinal strain (RVGLS), RV free wall strain (RVFWS) and left ventricular global longitudinal strain (LVGLS). During a median follow-up of 1,367 [959–2,123] days, 33 patients (33%) died. RVGLS was lower in non-survivors [−13.9% (−16.4 to −12.9)] than survivors [−17.1% (−20.2 to −15.2); *P* = 0.001]. In contrast, LVGLS as well as the conventional parameters LV ejection fraction (LVEF) and RV fractional area change (RVFAC) did not differ (*P* = ns). Kaplan–Meier analyses indicated a reduced survival probability when RVGLS was below the −14.6% cutpoint (*P* < 0.001). Lower RVGLS was associated with higher mortality [HR 1.13 (95% CI 1.04–1.23); *P* = 0.003] independent of LVGLS, LVEF, RVFAC, and EuroSCORE II. Addition of RVGLS clearly improved the fitness of bivariable and multivariable models including LVGLS, LVEF, RVFAC, and EuroSCORE II with potential incremental value for mortality prediction. In contrast, LVGLS, LVEF, and RVFAC were not associated with mortality.

**Discussion:**

In patients with severe AS undergoing TAVI, RVGLS but not LVGLS was reduced in non-survivors compared to survivors, differentiated non-survivors from survivors, was independently associated with mortality, and exhibited potential incremental value for outcome prediction. RVGLS appears to be more suitable than LVGLS for risk stratification in AS and timely valve replacement.

## Graphical Abstract

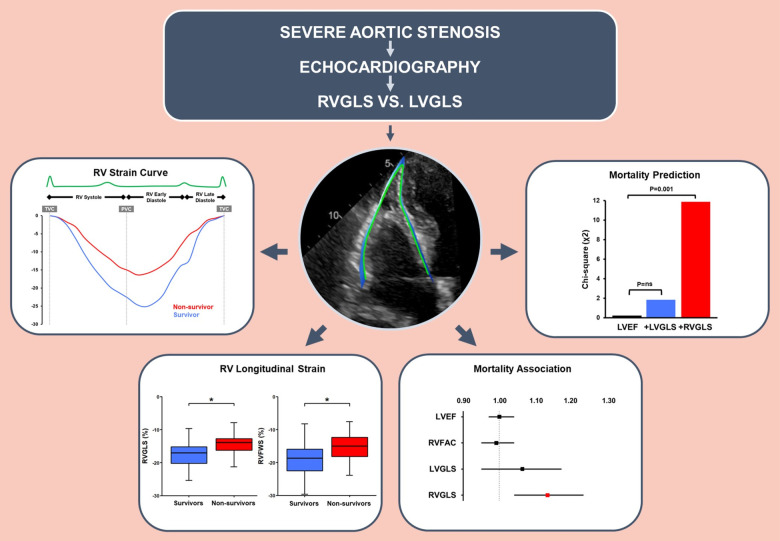

## 1. Introduction

Degenerative aortic stenosis (AS) is the most common valvular heart disease in high-income countries (1, 2). Myocardial remodelling does not only occur in the left (LV) but also the right ventricle (RV) of affected patients (3, 4). Chronic pressure overload triggers an adaptive response of the LV (5–7) eventually leading to functional and structural changes of the RV and ultimately resulting in impaired biventricular function with poor prognosis (3, 8).

Current guidelines recommend aortic valve replacement in severe symptomatic AS or in severe asymptomatic AS with associated LV systolic dysfunction defined as impaired LV ejection fraction (LVEF <50%) (2). The prognostic value of LVEF in severe AS is controversial, as it often deteriorates late in the disease course when permanent myocardial damage has occurred already (6, 9, 10). Hence, LVEF is an insensitive marker for early detection of LV dysfunction with questionable benefit for patient management aiming at preserving ventricular function. Speckle tracking echocardiography (STE) has become a clinically feasible method for assessing myocardial deformation (11–13). Global longitudinal strain may detect subclinical LV dysfunction (14–16) associated with reduced survival in AS incremental to other clinical (10, 15, 17) and echocardiographic parameters including LVEF (10, 13, 14, 15, 17, 18, 19).

Recent research highlights the importance of the RV for risk stratification in AS, but conventional echocardiographic parameters of RV function such as RV fractional area change (RVFAC), tricuspid annular plane systolic excursion (TAPSE), and RV lateral wall tissue velocity (*S'*) have shown inconsistent results regarding outcome association in patients undergoing transcatheter aortic valve implantation (TAVI) (20–23). Analysis of longitudinal strain in both ventricles offers new perspectives for risk stratification of patients with severe AS. A recent cardiac magnetic resonance (CMR) study demonstrated that RV global longitudinal strain (RVGLS) but not LV global longitudinal strain (LVGLS) predicted one-year mortality in patients undergoing TAVI (24). Similarly, an echocardiographic study in patients with low-flow low-gradient AS suggested that RVGLS has incremental prognostic value compared to LVGLS (25).

This study aims at exploring the role of RVGLS vs. LVGLS assessed by STE for outcome prediction in a representative patient cohort with severe AS undergoing TAVI.

## 2. Materials and methods

### 2.1. Study population

One hundred patients with severe AS (aortic valve area <1 cm^2^ or indexed AVA <0.6 cm^2^/m^2^) undergoing TAVI between 2008 and 2019 were retrospectively identified from the prospective AS registry of the University Heart Center Zurich. Patients were included when a comprehensive echocardiographic examination was available within three months prior to TAVI allowing complete strain analysis of both ventricles. A flow diagram (Supplementary Figure S1) illustrates how patients were enrolled for the study. Ethical committee approval and informed consent were obtained prior to patient inclusion.

### 2.2. Echocardiography and strain analysis

Transthoracic echocardiographic (TTE) examinations were performed using commercially available equipment (iE33 or Epiq 7, Philips Medical Systems, The Netherlands; E9 or E95, GE Healthcare, USA). Echocardiographic measurements were made by certified specialists according to current recommendations (26–28). Calculation of LVEF was based on Simpson's biplane method.

TomTec ImageArena Cardiac Performance Analysis (Version 4.6) was used for offline STE measurements of both ventricles according to current recommendations (26, 27). Endocardial tracing was performed manually excluding sigmoid septal hypertrophy, papillary muscles, and trabeculations. Heart cycle timing was identified from the M-mode.

Apical 2- (A2C), 3- (A3C) and 4-chamber (A4C) views were used for measuring LVGLS. End-diastole was set at the last frame before mitral valve closure and end-systole at the last frame before aortic valve closure. LVGLS is indicated as average peak systolic strain based on the 16-segment model. Focused RV views were used for measuring RV strain (29). End-diastole was defined as the last frame before tricuspid valve closure and end-systole as the smallest ventricular systolic dimension, respectively. RVGLS is indicated as average peak systolic strain based on the 6-segment model and RV free wall strain (RVFWS) on the 3 segments of the free wall, respectively.

### 2.3. Reproducibility of strain measurements

Reproducibility was tested on 15 echocardiographic examinations by two observers to investigate inter-observer agreement and repeated by the main observer with a difference of 3 months between the first and the second measurement to determine intra-observer agreement. Concordance correlation coefficient was used for assessing reproducibility (inter-observer agreement) and repeatability (intra-observer agreement). These results are reported in Supplementary Table S1. There is strong inter- and intra-observer agreement for LV and RV strain measurements.

### 2.4. Follow-up

The date of the echocardiography examination before TAVI (i.e., within 3 months prior to procedure) marks the date of study inclusion. The date of the TAVI procedure indicates the start of follow-up. All-cause mortality was defined as the primary endpoint. Patient survival status was evaluated through patient records and/or phone calls.

### 2.5. Statistics

The Shapiro–Wilk test was used to assess normal distribution, with most variables displaying a non-normal distribution. Continuous variables are given as median [interquartile range, IQR] and categorical variables as absolute number (percentage). Continuous variables were compared with the Mann–Whitney–Wilcoxon test, categorical variables with Fisher's exact test. Receiver operating characteristic (ROC) curve analysis was used to determine the optimal cutpoint value for distinguishing survivors from non-survivors, and model discrimination was summarised by area under the curve (AUC). The AUC from models was compared with the DeLong method using *pROC* (version 1.18.0) package. Kaplan–Meier survival curves and log-rank tests of time-to-event data were analysed with the *survminer* (version 0.4.9) package; variables were either dichotomised at their optimal cutpoint according to the ROC curve or according to the literature. For some analyses, variables were divided into tertiles or quartiles. Association with all-cause mortality was analysed in uni- and multivariable Cox regression models. Proportional hazard assumptions were assessed for all models using the scaled Schoenfeld residuals. Variables with clinical relevance such as age, sex, EuroSCORE II, STS score, AS severity, LVEF, RVFAC were included in multivariable models regardless of their significance level in univariable models and tested for sensitivity. A further sensitivity analysis was performed excluding periprocedural deaths from the total number of events. Cox regression analysis of variance (Cox-ANOVA) was used to test model fit. The chi-squared (*χ*^2^) log-likelihood ratio and Harrell's C-statistic were used to examine the incremental value of predictors in the multivariable model compared to the nested univariable model. Collinearity between variables in the regression models was tested using Spearman's correlation and variance inflation factor tests. Standard mean difference (SMD) was used for determining the representativeness of the study cohort compared to the overall registry population. SMD values of 0.2, 0.5, and 0.8 are considered to represent small, medium, and large differences, respectively (30, 31). Statistical analyses were performed using MedCalc® version 19.6.4 and R version 4.1.3. Statistical significance was considered at a two-sided *P* value <0.05.

## 3. Results

### 3.1. Baseline characteristics

Tables 1, 2 summarise the clinical and echocardiographic baseline characteristics, respectively. Almost all patients exhibited normal size and systolic function of both ventricles as determined by RV enddiastolic area index (RVEDAI), RVFAC, TAPSE, left ventricular enddiastolic volume index (LVEDVI), and LVEF. There was little difference between the baseline characteristics of the study cohort and those of the overall registry population (*N* = 1,467; SMD 0.0–0.4; Supplementary Table S2). The study cohort was slightly younger and exhibited a lower EuroSCORE II, while mean transaortic pressure gradient (MTPG) was slightly higher, AVA was similar, and RVFAC, TAPSE, LVEF were marginally higher (Supplementary Table S2).

**Table 1:** Clinical baseline characteristics.

Parameters	Overall (*N* = 100)	Survivors (*N* = 67)	Non-survivors (*N* = 33)	*P*
Age, years	79.0 [75.8–84.0]	79.0 [74.5–83.0]	81.0 [77.0–84.0]	0.164
Women, *N* (%)	49 (49)	32 (48)	17 (52)	0.888
BMI, kg/m^2^	26.6 [24.2–30.2]	26.3 [24.0–29.9]	27.3 [25.2–30.8]	0.221
BSA, m^2^	1.8 [1.7–1.9]	1.8 [1.7–2.0]	1.9 [1.7–1.9]	0.716
Hypertension, *N* (%)	77 (77)	50 (75)	27 (82)	0.582
Diabetes, *N* (%)	36 (36)	23 (34)	13 (39)	0.784
Dyslipidaemia, *N* (%)	68 (68)	46 (69)	22 (67)	0.554
Clinically relevant CAD, *N* (%)	57 (57)	40 (60)	17 (52)	0.574
CABG, *N* (%)	16 (16)	10 (15)	6 (18)	0.923
PAD, *N* (%)	17 (17)	11 (16)	6 (18)	0.925
Cerebrovascular disease, *N* (%)	24 (24)	17 (25)	7 (21)	0.523
NYHA III or IV, *N* (%)	8 (8)	7 (11)	1 (3)	0.372
COPD, *N* (%)	13 (13)	8 (12)	5 (15)	0.560
eGFR, ml/min/1.73 m^2^	61.0 [48.0–73.5]	62.0 [50.0–75.5]	55.1 [39.0–69.0]	0.262
Atrioventricular block III, N (%)	1 (1)	1 (2)	0 (0)	0.210
Ventricular conduction abnormality, *N* (%)				0.005*
BFB	3 (3)	2 (3)	1 (3)	
LAHB	7 (7)	2 (3)	5 (15)	
LBBB	7 (7)	7 (11)	0 (0)	
RBBB	11 (11)	4 (6)	7 (21)	
EuroSCORE II, %	3.0 [1.7–4.9]	2.9 [1.5–4.2]	3.2 [1.8–5.7]	0.169
Periprocedural death, *N* (%)	3 (3)	0 (0)	3 (9)	0.055

BMI, body mass index; BSA, body surface area; CAD, coronary artery disease; CABG, coronary artery bypass graft; PAD, peripheral arterial disease; NYHA, New York Heart Association; COPD, chronic obstructive pulmonary disease; eGFR, estimated glomerular filtration rate; BFB, bifascicular block; LAHB, left anterior hemiblock; LBBB, left bundle branch block; RBBB, right bundle branch block.

Values are given as median (IQR, interquartile range) for continuous variables or number (percentage) for catecorical variables.

* Significant values (*P* < 0.05).

**Table 2:** Echocardiographic baseline characteristics.

Parameters	Overall (*N* = 100)	Survivors (*N* = 67)	Non-survivors (*N* = 33)	*P*
Aortic stenosis severity				
MTPG, mmHg	43.0 [35.0–52.0]	43.0 [34.5–50.5]	44.0 [38.0–56.0]	0.257
AVA, cm^2^	0.8 [0.7–0.9]	0.8 [0.7–0.9]	0.8 [0.6–0.9]	0.304
AVAI, cm^2^/m^2^	0.4 [0.4–0.5]	0.4 [0.4–0.5]	0.4 [0.4–0.5]	0.126
Conventional				
RVEDAI, cm^2^/m^2^	9.2 [8.0–10.7]	9.1 [7.8–11.0]	9.4 [8.4–10.5]	0.913
RVFAC, %	40.5 [37.8–44.0]	41.0 [38.0–44.0]	40.0 [33.0–45.0]	0.537
TAPSE, mm	20.0 [18.0–22.5]	20.0 [18.0–22.0]	20.0 [17.0–23.0]	0.626
LVEF, %	58.5 [53.0–64.3]	58.0 [53.5–64.0]	60.0 [53.0–65.0]	0.657
LVEDVI, ml/m^2^	53.0 [41.8–65.0]	54.0 [44.0–65.5]	51.0 [38.0–63.0]	0.328
LVSVI, ml/m^2^	40.5 [32.3–48.0]	42.0 [35.0–48.0]	37.0 [27.0–48.0]	0.111
LVMMI, g/m^2^	101.5 [86.8–122.3]	99.0 [88.5–116.5]	107.0 [76.0–151.0]	0.224
Speckle-tracking				
RVGLS, %	−16.5 [−19.8– −13.8]	−17.1 [−20.2– −15.2]	−13.9 [−16.4– −12.9]	0.001*
RVFWS, %	−17.6 [−21.6– −14.3]	−18.7 [−22.6– −15.9]	−15.7 [−18.3– −12.8]	0.002*
LVGLS, %	−10.5 [−13.0– −8.4]	−10.8 [−13.1– −8.7]	−10.3 [−12.5– −6.8]	0.303

MTPG, mean transaortic pressure gradient; AVA, aortic valve area; AVAI, aortic valve area index; RVEDAI, right ventricular end diastolic area index; RVFAC, right ventricular fractional area change; TAPSE, tricuspid annular plane systolic excursion; LVEF, left ventricular ejection fraction; LVEDVI, left ventricular end-diastolic volume index; LVSVI, left ventricular stroke volume index; LVMMI, left ventricular muscle mass index; RVGLS, right ventricular global longitudinal strain; RVFWS, right ventricular free wall strain; LVGLS, left ventricular global longitudinal strain.

Values are given as median (IQR, interquartile range).

* Significant values (*P* < 0.05).

### 3.2. Ventricular systolic function

**Figure 1:**
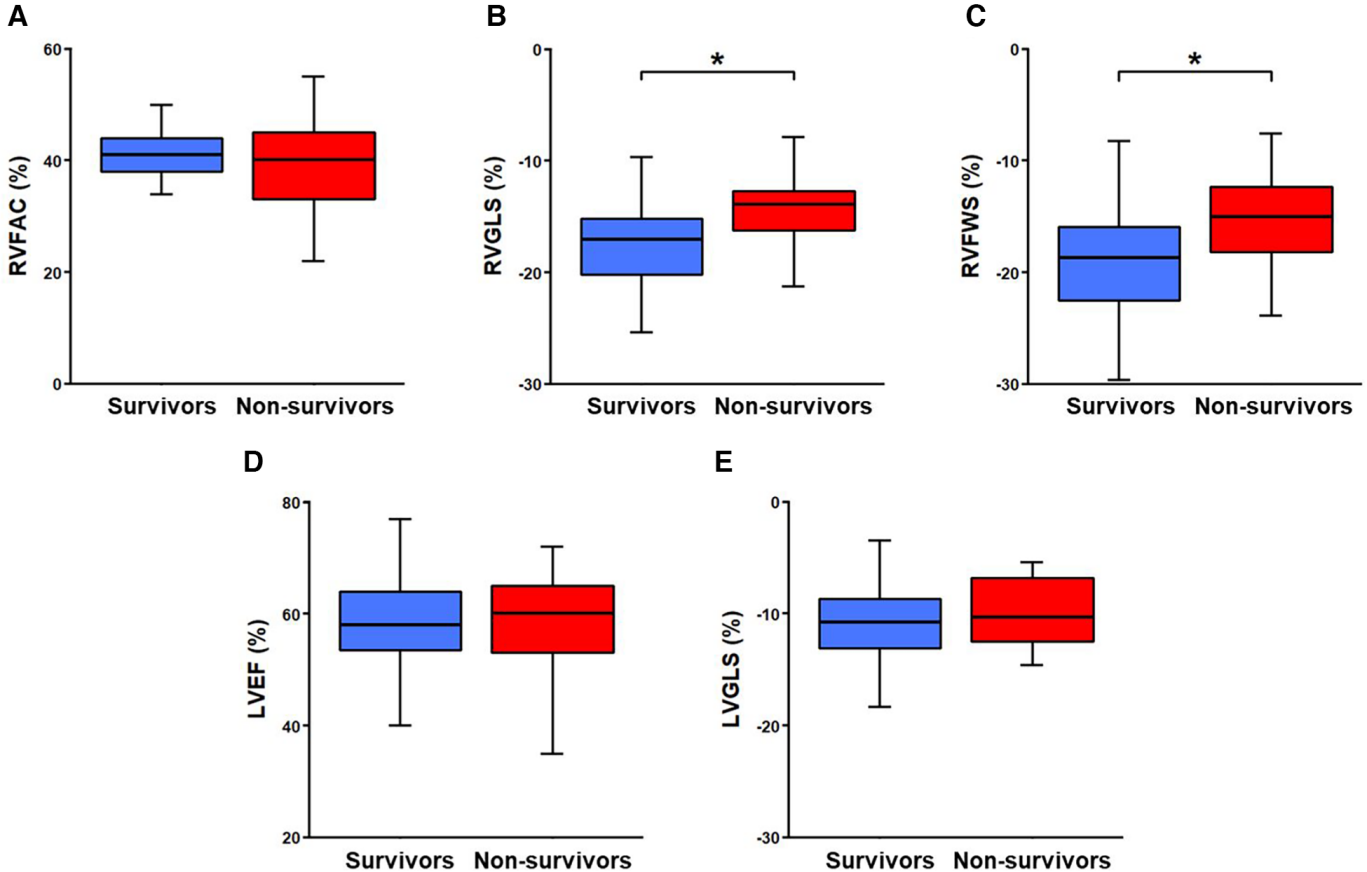
Echocardiographic parameters dichotomised by survival status. RVFAC, right ventricular fractional area change (panel **A**); RVGLS, right ventricular global longitudinal strain (panel **B**); RVFWS, right ventricular free wall strain (panel **C**); LVEF, left ventricular ejection fraction (panel **D**); LVGLS, left ventricular global longitudinal strain (panel **E**). Mann–Whitney–Wilcoxon test was used for comparison of continuous variables within groups. *Significant values (*P* < 0.05).

RVFAC [40.5% (37.8–44.0)] and LVEF [58.5% (53.0–64.3)] were preserved among the study population. In contrast, longitudinal deformation of both ventricles was impaired (RVGLS [−16.5% (−19.8 to −13.8)]; RVFWS (−17.6% [−21.6 to −14.3]; LVGLS [−10.5% (−13.0 to −8.4); Figure 1].

The concordance correlation coefficient for assessing intra- and inter-observer variability was each 0.8 for LVGLS, each 0.9 for RVGLS, and 0.9 and 0.8 for RVFWS, respectively (Supplementary Table S1).

### 3.3. Survival

**Figure 2:**
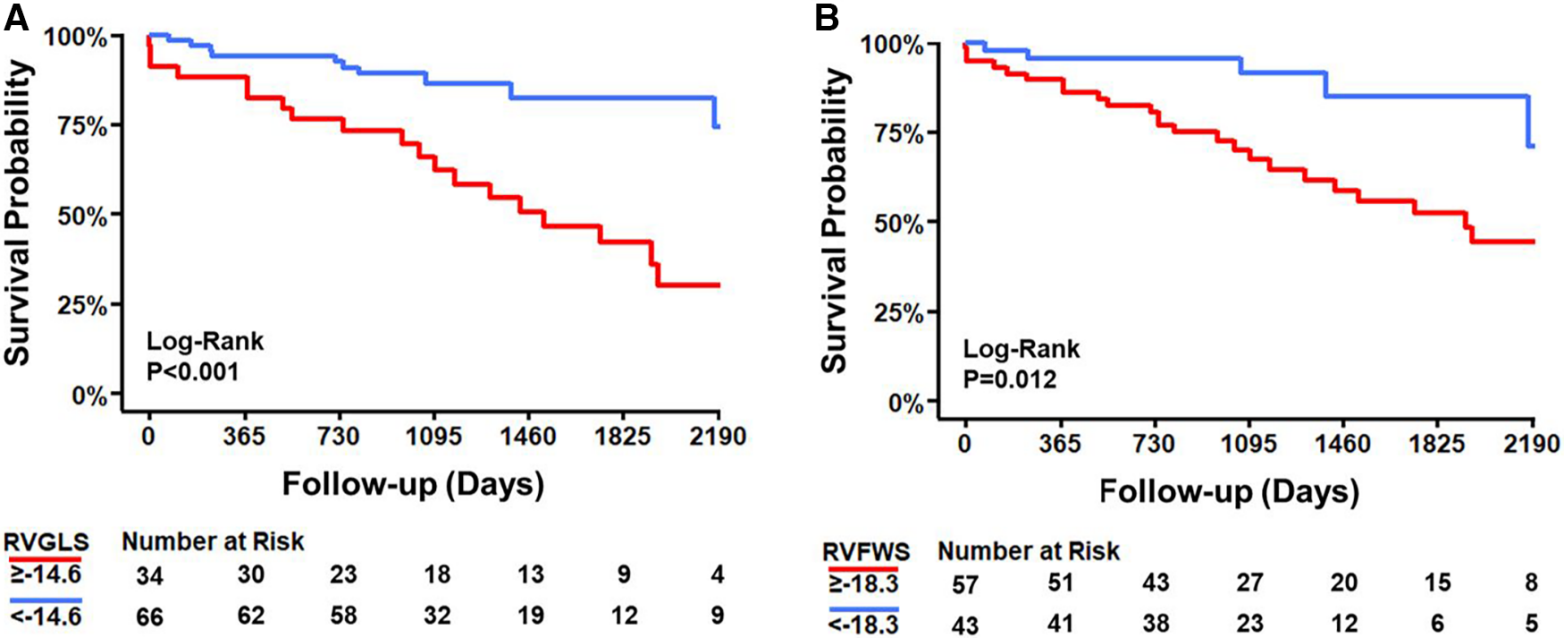
Kaplan–Meier survival curves differentiating survivors from non-survivors with the cutpoints for right ventricular global longitudinal strain (RVGLS; panel **A**) and right ventricular free wall strain (RVFWS; panel **B**).

During a median follow-up time of 1,367 [959–2,123] days, 33 patients (33%) died, of which 23 (23%) due to a cardiovascular cause. There was no significant difference in survival between women and men (Table 1). Among the STE-derived parameters, RVGLS and RVFWS were significantly lower in non-survivors (−13.9% [−16.4 to −12.9]; *P* = 0.001 and −15.7% [−18.3 to −12.8]; *P* = 0.002, respectively) compared to survivors (−17.1% [−20.2 to −15.2]; *P* = 0.001 and −18.7% [−22.6 to −15.9]; *P* = 0.002, respectively), while LVGLS did not differ (*P* = 0.303; Table 2). The cutpoint values for RVGLS (≥−14.6%; sensitivity 61%; specificity 79%; ROC AUC 70%; *P* < 0.001) and RVFWS (≥−18.3%; sensitivity 79%; specificity 54%; ROC AUC 69%; *P* = 0.001) differentiated survivors from non-survivors, while that for LVGLS did not (*P* = 0.243). Kaplan–Meier analyses indicated a higher survival probability when the population was dichotomised according to the cutpoint value for RVGLS (*P* < 0.001) and for RVFWS (*P* = 0.012; Figure 2), but not for LVGLS (*P* = 0.580).

**Figure 3:**
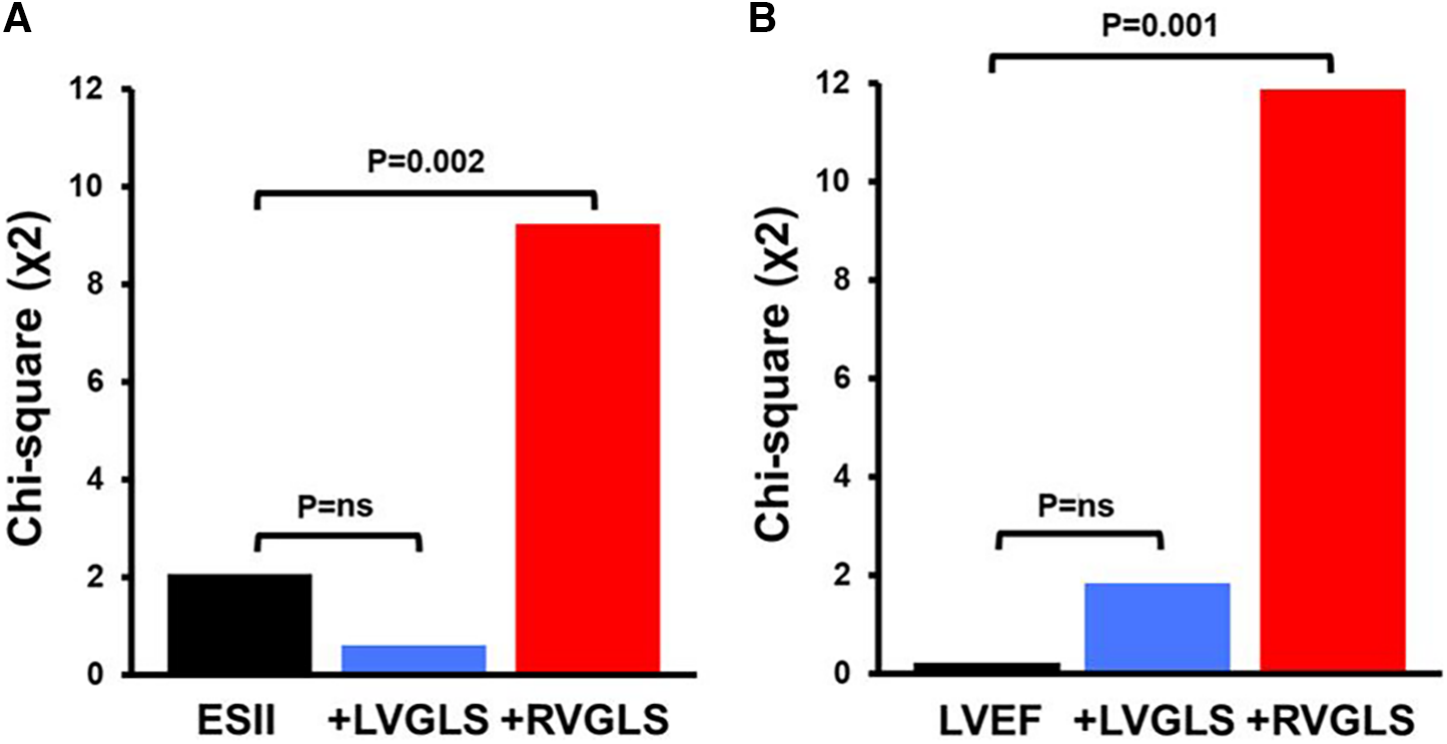
Incremental prognostic value of right ventricular global longitudinal strain (RVGLS) over baseline risk factors represented by EuroSCORE II (ESII; panel A) or left ventricular ejection fraction (LVEF; panel B); ns, non significant.

Univariable Cox regression analysis associated a lower RVGLS [HR 1.13 (95% CI 1.04–1.23); *P* = 0.003; ANOVA χ^2^ 9.27; χ^2^ *P* = 0.002] and RVFWS (data not shown) with an increased mortality risk, while this was not the case for LVGLS [HR 1.06 (0.95–1.17); *P* = 0.300; ANOVA χ^2^ 1.09; χ^2^ *P* = 0.297] or the conventional echocardiographic parameters (Table 3). Bivariable Cox regression analysis demonstrated that the association of RVGLS and RVFWS with mortality remained independent of LVGLS or other clinically relevant covariables such as RVFAC, LVEF, and EuroSCORE II (Table 3; RVFWS data not shown). Likelihood ratios showed that inclusion of RVGLS or RVFWS to these models significantly improved their fitness with potential incremental value for association with mortality, while LVGLS did not (Table 3; Figure 3; RVFWS data not shown). The greatest C-index was observed for the univariable RVGLS model and the bivariable model containing RVGLS and LVGLS [both 0.69 (0.58–0.80)].

**Table 3:** Univariable and bivariable Cox regression models.

Nested Model Bivariable Model	Cox Regression			Model Fit		Harrell’s C-statistic	
	HR	95% CI	*P*	χ^2^	χ^2^ *P*	C-index	95% CI
RVGLS	1.13	1.04–1.23	0.003*	9.27	0.002*	0.69	0.58–0.80
RVGLS LVGLS	1.14 0.98	1.04–1.25 0.86–1.11	0.007* 0.720	0.13	0.717	0.69	0.58–0.80
LVGLS	1.06	0.95–1.17	0.300	1.09	0.297	0.58	0.47–0.70
LVGLS RVGLS	0.98 1.14	0.86–1.11 1.04–1.25	0.720 0.007*	8.31	0.004*	0.69	0.58–0.80
RVFAC	0.99	0.95–1.04	0.660	0.18	0.670	0.52	0.39–0.66
RVFAC RVGLS	1.03 1.17	0.98–1.07 1.06–1.29	0.250 0.002*	10.45	0.001*	0.68	0.57–0.79
RVFAC LVGLS	1.00 1.06	0.95–1.05 0.94–1.18	0.970 0.340	0.91	0.341	0.58	0.47–0.70
LVEF	1.00	0.97–1.04	0.850	0.03	0.853	0.46	0.34–0.57
LVEF RVGLS	1.03 1.18	0.99–1.06 1.06–1.30	0.120 0.001*	11.87	0.001*	0.67	0.55–0.78
LVEF LVGLS	1.02 1.09	0.98–1.05 0.97–1.22	0.380 0.170	1.83	0.176	0.56	0.44–0.68
LVEF RVFAC	1.01 0.98	0.97–1.04 0.93–1.04	0.690 0.570	0.32	0.574	0.50	0.37–0.63
EuroSCORE II	1.06	0.99–1.15	0.110	2.08	0.149	0.55	0.43–0.67
EuroSCORE II RVGLS	1.06 1.13	0.98–1.15 1.04–1.22	0.120 0.003*	9.22	0.002*	0.66	0.54–0.78
EuroSCORE II LVGLS	1.06 1.04	0.98–1.14 0.94–1.16	0.170 0.430	0.61	0.435	0.56	0.44–0.68
EuroSCORE II RVFAC	1.06 0.99	0.98–1.15 0.95–1.04	0.120 0.710	0.13	0.719	0.52	0.39–0.65

HR, hazard ratio; CI, confidence interval; χ^2^, chi-square. RVGLS, right ventricular global longitudinal strain; LVGLS, left ventricular global longitudinal strain; RVFAC, right ventricular fractional area change; LVEF, left ventricular ejection fraction.

Association with mortality in univariable (nested model) and bivariable Cox regression analysis. RVGLS showed incremental value over baseline LVGLS, conventional echocardiographic parameters, and clinical parameters as represented by EuroSCORE II.

* Significant values (*P* < 0.05).

A multivariable Cox regression analysis was performed to test for a possible interaction between LVGLS and RVGLS or RVFWS, respectively, with inclusion of EuroSCORE II to account for possible confounders (Table 4). RVGLS [1.15 (1.04–1.26); *P* = 0.005] and RVFWS [1.14 (1.05–1.25); *P* = 0.003] were associated with mortality independent of LVGLS and EuroSCORE II. There was a significant interaction between RVGLS and LVGLS regarding the association with mortality [0.97 (0.95–1.00); *P* = 0.043]. In contrast, RVFWS and LVGLS did not interact significantly in terms of association with mortality [0.98 (0.96–1.01); *P* = 0.210]. The highest C-index [0.69 (0.58–0.80)] of the multivariable models was observed for the model containing RVFWS, LVGLS, their interaction, and the EuroSCORE II. However, this is no higher than the greatest univariable C-index and there were only small differences in C-index between the multivariable models. Finally, a sensitivity analysis was performed by replacing EuroSCORE II with either STS score, age and sex, or parameters defining AS severity (i.e., MTPG, AVA), revealing most compelling results for models including EuroSCORE II (data not shown).

**Table 4:** Multivariable Cox regression models.

Variables	Cox Regression			Model Fit		Harrells’c C-statistic	
	HR	95% CI	*P*	χ^2^	χ^2^ *P*	C-index	95% CI
EuroSCORE II RVGLS LVGLS	1.07 1.15 0.95	1.00–1.16 1.04–1.26 0.83–1.09	0.055 0.005* 0.470			0.66	0.54–0.79
EuroSCORE II RVGLS LVGLS RVGLS:LVGLS	1.08 1.21 1.00 0.97	1.00–1.17 1.08–1.36 0.87–1.15 0.95–1.00	0.059 0.001* 0.950 0.043*	4.85	0.028*	0.68	0.58–0.79
EuroSCORE II RVFWS LVGLS	1.05 1.14 0.96	0.97–1.14 1.05–1.25 0.85–1.09	0.19 0.003* 0.570			0.68	0.56–0.80
EuroSCORE II RVFWS LVGLS RVFWS:LVGLS	1.06 1.16 1.00 0.98	0.98–1.15 1.06–1.27 0.88–1.14 0.96–1.01	0.160 0.001* 0.990 0.210	1.66	0.198	0.69	0.58–0.81

HR, hazard ratio; CI, confidence interval; χ^2^, chi–square. RVGLS, right ventricular global longitudinal strain; LVGLS, left ventricular global longitudinal strain; RVFWS, right ventricular free wall strain.

Association with mortality in multivariable Cox regression analysis including interaction between LVGLS and RVGLS or RVFWS, respectively. RV longitudinal strain showed incremental value over LVGLS and clinical parameters as represented by EuroSCORE II.

* Significant values (*P* < 0.05).

## 4. Discussion

This study demonstrates that in patients with severe AS undergoing TAVI (1) RV strain was lower in non-survivors than survivors; (2) RV strain was associated with mortality after TAVI; (3) RV strain was an independent predictor of mortality; and (4) RV strain improved the fitness of bivariable and multivariable models with potential incremental value for association with mortality. In contrast, none of the LV parameters was associated with mortality nor useful for predicting outcome in this cohort.

The present data indicate that RVGLS, but not LVGLS, is an independent predictor of mortality in patients with severe AS undergoing TAVI. Previous studies observed that pre-interventional RV strain plays a role in predicting outcomes in such patients (19, 23, 32). Those studies focused on RV strain without including LV deformation and exhibited a relatively short follow-up (23, 32) or small study population (19). To the best of our knowledge, the role of RVGLS vs. LVGLS has not been investigated by STE for predicting outcomes of patients with severe AS undergoing TAVI. A previous echocardiographic study reported an incremental prognostic value of RVFWS over LVGLS (25). In contrast to the present work, that study investigated patients with low-flow low-gradient AS undergoing surgical aortic valve replacement or conservative management and found a reduced survival associated with lower RVFWS in both groups (25). Similar to our study, appropiate RV views seemed to be a limitating factor (25). A recent CMR study showed that RVFWS, but not LVGLS, predicted 1-year all-cause mortality in patients with severe AS undergoing TAVI (24). The present findings are consistent with that observation, with STE having the advantages of lower cost, wider availability, and routine application in the pre-interventional assessment as compared to CMR. Finally, given the rather small but nevertheless representative cohort in the current study, neither definition criteria for severe AS nor parameters of ventricular function were compared for the representativeness analyses in that study (24). Similar findings were observed in patients with heart failure. RV strain measured by STE was independently associated with mortality (33), had a higher predictive value for mortality than LVGLS (34), was the main determinant of myocardial fibrosis (35) and showed good agreement with strain measurements by CMR in these patients (36).

LVGLS almost invariably exhibited clearly reduced values in the present cohort, regardless of survival status during follow-up, and did not even tend to be associated with mortality. This observation is consistent with some reports (24, 37, 38), but is contrary to other published literature (10, 11, 14, 16, 17, 25, 37, 38, 39, 40). In the present representative cohort with AS, all patients exhibited severe valvular heart disease and qualified for TAVI. The low median LVGLS with low variability in the population may indicate advanced cardiac disease due to long-standing increase in ventricular afterload. Adaptive cardiac remodelling seems to affect the LV in a rather uniform manner across the population while reaching the RV in part of the latter only. In line with this interpretation, RV dysfunction may reflect more advanced cardiac remodelling, and its sustained outcome association after TAVI suggests that remodelling may have reached an irreversible stage. In addition to different stages of cardiac remodelling in response to AS, higher values for and/or larger variability of LVGLS may occur due to differences in study design or study population such as specific subsets of AS (17, 37, 39), different disease management (15, 16, 17, 37, 38, 41), different follow-up duration (14, 15), or different endpoint definition (17, 38).

RVFAC, TAPSE, and LVEF were not associated with outcome in the present cohort, which is consistent with previous observations and emphasises that longitudinal deformation as well as other strain-derived parameters such as ventricular twist are much more sensitive tools for detecting ventricular remodelling (14, 18, 42). The LV remodelling response indeed affects the myocardium gradually from the subendocardial to the subepicardial layer, with longitudinal, circumferential, and radial function being affected in corresponding order (12). As a result, LVEF may be preserved despite the progressive increase in ventricular afterload and the deranged longitudinal function (43). The RV exhibits a similar albeit simpler myocardial structure with fibres oriented longitudinally in the subendocardium and circumferentially in the subepicardium, respectively, provoking alterations in longitudinal strain vs. conventional parameters analogous to those occurring in the LV (44).

Timely assessment of RV longitudinal strain seems to be crucial for recognising the extent of myocardial remodelling in response to AS. Appropriate clinical decision-making early in the disease course may decrease the risk of irreversible myocardial damage and improve survival after aortic valve replacement.

### 4.1. Clinical implications

Assessment of RV systolic function is an integral part of every echocardiographic examination. RV strain is obtained from the RV focused apical view with little additional effort. However, care must be taken in the real-wold setting to ensure that all methodological requirements are met in order to perform a reliable deformation analysis. Impaired RV longitudinal strain indicates increased mortality in patients with severe AS after valve replacement and emerges as an important parameter to improve the prognostic understanding of these patients. Hence, RV longitudinal strain should be measured in patients with severe AS and its reduced values should promote the decision to replace the aortic valve for avoiding persistent cardiac remodelling and reduced outcome after intervention.

### 4.2. Limitations

This study is limited by its retrospective single-center design; however, the cohort is representative of a typical TAVI population and the number of patients included, although limited, is reasonable to address the research question. A selection bias cannot be excluded because inclusion criteria involved the quality of echocardiographic examinations as well as several additional parameters. Although multivariable models were generated, it cannot be excluded completely that unobserved confounding factors affected the results. The predictive performance, as measured by AUC, was greater for the parameters of interest than for EuroSCORE II, which contains several predictors important to understanding individual prognosis. Due to the limited amount of data, the EuroSCORE II was used as a single predictor and its individual components were not re-estimated with the data at hand, tipping the comparison in favor of the new parameters. More data on this population is needed to develop true clinical prediction models with limited risk of overfitting and performance overoptimism.

## 5. Conclusion

In patients with severe AS undergoing TAVI, RV longitudinal strain was lower in non-survivors than survivors and independently associated with mortality after TAVI. RV longitudinal strain improved the fitness of clinical and echocardiographic models with potential incremental value for mortality prediction. LVGLS was not associated with mortality in the current dataset. Hence, RV longitudinal strain may be incorporated in risk stratification of patients with severe AS and trigger timely aortic valve replacement.

## Data Availability

The raw data supporting the conclusions of this article will be made available by the authors, without undue reservation.
