# Supplementary material for: Right vs. left ventricular longitudinal strain for mortality prediction after transcatheter aortic valve implantation
Source: Front Cardiovasc Med. 2023 Sep 7;10:1252872. doi: 10.3389/fcvm.2023.1252872 (PMC10513390; doi:10.3389/fcvm.2023.1252872)
Supplement: Supplementary file 1 [file Datasheet1.docx]

Supplementary Material

**Supplementary Table S1.** Reproducibility of strain measurements.

| **Parameters (*N* = 15)** | **Concordance Correlation Coefficient** | **95% CI** |
| --- | --- | --- |
| **Inter-observer Agreement** | |  |
| **RVGLS, %** | **0.89** | **0.75–0.95** |
| **RVFWS, %** | **0.79** | **0.52–0.91** |
| **LVGLS, %** | **0.78** | **0.48–0.91** |
| **Intra-observer Agreement** | |  |
| **RVGLS, %** | **0.87** | **0.66–0.95** |
| **RVFWS, %** | **0.85** | **0.61–0.95** |
| **LVGLS, %** | **0.80** | **0.54–0.92** |

Reproducibility (inter-observer agreement) and repeatability (intra-observer agreement) of strain measurements showing a strong inter- and intra-observer agreement for RV and LV strain measurements. CI, confidence interval.

RVGLS, right ventricular global longitudinal strain; RVFWS, right ventricular free wall strain; LVGLS, left ventricular global longitudinal strain; RVFWS, right ventricular free wall strain.

**Supplementary Table S2.** Representativeness of study cohort.

| **Parameters** | **Overall AS Registry**  **(*N* = 1467)** | **Excluded**  **(*N* = 1367)** | **Study Population**  **(*N* = 100)** | **SMD** |
| --- | --- | --- | --- | --- |
| **Age, years** | **80.9 (7.3)** | **81.0 (7.3)** | **78.9 (6.9)** | **0.292** |
| **Men, *N* (%)** | **764 (52)** | **713 (52)** | **51 (51)** | **0.028** |
| **BMI, kg/m^2^** | **26.9 (4.8)** | **26.9 (4.8)** | **27.5 (4.9)** | **0.137** |
| **BSA, m^2^** | **1.8 (0.2)** | **1.8 (0.2)** | **1.8 (0.2)** | **0.017** |
| **Clinically relevant CAD, *N* (%)** | **839 (57)** | **782 (57)** | **57 (57)** | **0.015** |
| **CABG, *N* (%)** | **286 (20)** | **270 (20)** | **16 (16)** | **0.102** |
| **Atrioventricular block, *N* (%)** | **3 (<1)** | **2 (<1)** | **1 (1)** | **0.275** |
| **AVB II** | **1 (<1)** | **1 (<1)** | **0 (0)** |  |
| **AVB III** | **2 (<1)** | **1 (<1)** | **1 (1)** |  |
| **Ventricular conduction abnormality, *N* (%)** | **439 (30)** | **411 (30)** | **28 (28)** | **0.378** |
| **BFB** | **40 (3)** | **37 (3)** | **3 (3)** |  |
| **LAHB** | **102 (7)** | **95 (7)** | **7 (7)** |  |
| **LBBB** | **119 (8)** | **112 (8)** | **7 (7)** |  |
| **RBBB** | **101 (7)** | **90 (7)** | **11 (11)** |  |
| **Pacing** | **77 (5)** | **77 (6)** | **0 (0)** |  |
| **EuroSCORE II, %** | **5.4 (4.8)** | **5.5 (4.8)** | **4.3 (4.8)** | **0.243** |
| **Aortic stenosis severity** |  |  |  |  |
| **MTPG, mm Hg** | **41.7 (15.8)** | **41.4 (15.9)** | **45.9 (14.3)** | **0.239** |
| **AVA, cm^2^** | **0.8 (0.2)** | **0.8 (0.2)** | **0.8 (0.2)** | **0.083** |
| **AVAI, cm^2^/m^2^** | **0.4 (0.1)** | **0.4 (0.1)** | **0.4 (0.1)** | **0.134** |
| **Echocardiography** |  |  |  |  |
| **RVFAC, %** | **38.9 (7.7)** | **38.7 (7.9)** | **40.1 (6.8)** | **0.183** |
| **TAPSE, mm** | **19.1 (4.7)** | **19.0 (4.8)** | **20.0 (3.9)** | **0.246** |
| **LVEF, %** | **54.2 (13.3)** | **54.0 (13.4)** | **57.0 (10.8)** | **0.246** |

Representativeness of the study cohort compared to the total registry population. Values are given as mean (SD, standard deviation) or number (percentage). Standard mean difference (SMD) was used for comparing the groups. SMD values of 0.2, 0.5, and 0.8 represent small, medium, and large differences, respectively (30, 31).

BMI, body mass index; BSA, body surface area; CAD, coronary artery disease; CABG, coronary artery bypass graft; BFB, bifascicular block; LAHB, left anterior hemiblock; LBBB, left bundle branch block; RBBB, right bundle branch block; MTPG, mean transaortic pressure gradient; AVA, aortic valve area; AVAI, aortic valve area index; RVFAC, right ventricular fractional area change; TAPSE, tricuspid annular plane systolic excursion; LVEF, left ventricular ejection fraction.

**
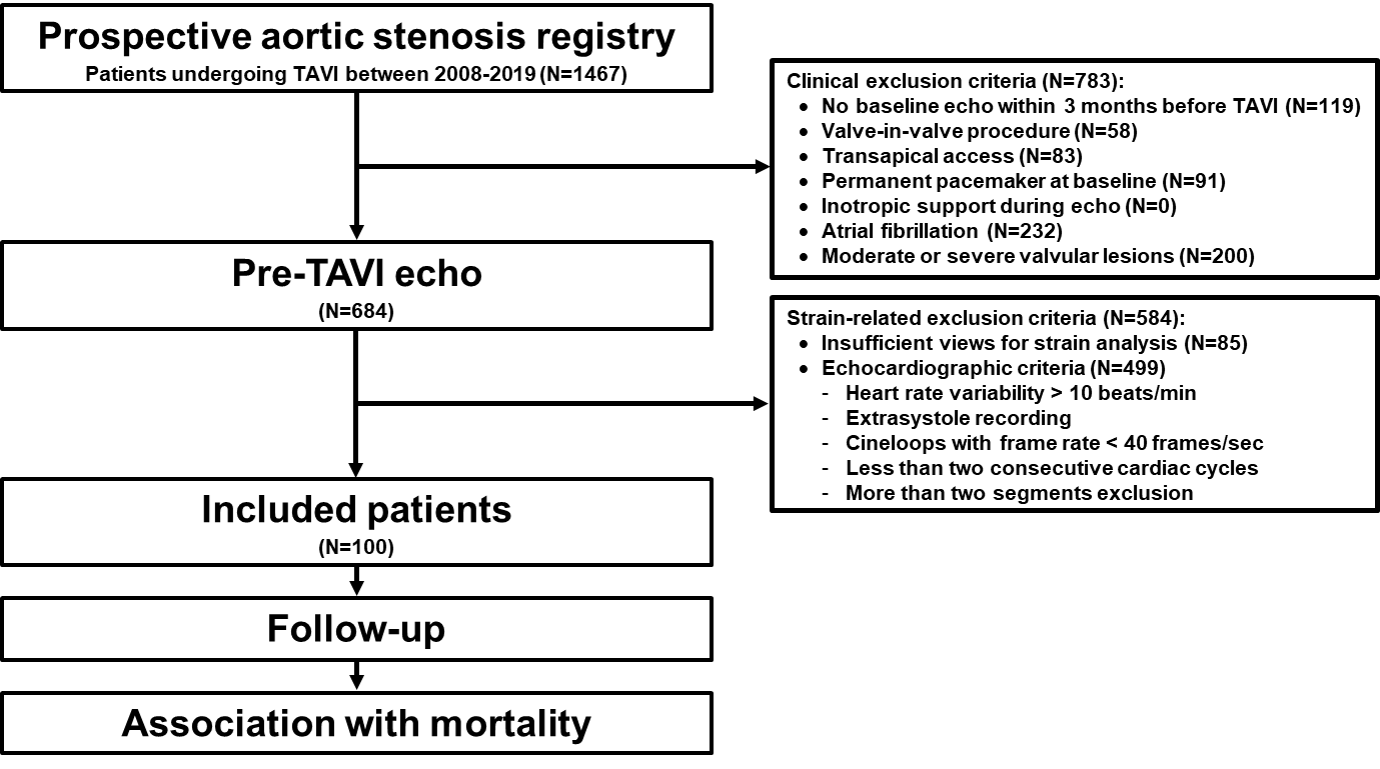
**

**Supplementary Figure S1.** Study flow diagram.

TAVI, transcatheter aortic valve implantation.
